# Neuroprotective therapies in glaucoma: II. Genetic nanotechnology tools

**DOI:** 10.3389/fnins.2015.00355

**Published:** 2015-10-14

**Authors:** Nafiseh Nafissi, Marianna Foldvari

**Affiliations:** School of Pharmacy and Waterloo Institute of Nanotechnology, University of WaterlooWaterloo, ON, Canada

**Keywords:** neurodegenerative disease, glaucoma, neurotrophic factor therapy, gene and cell therapy, nanotechnology, minicircle DNA vectors, transposon systems, ZFN- TALEN-CRISPR/Cas

## Abstract

Neurotrophic factor genome engineering could have many potential applications not only in the deeper understanding of neurodegenerative disorders but also in improved therapeutics. The fields of nanomedicine, regenerative medicine, and gene/cell-based therapy have been revolutionized by the development of safer and efficient non-viral technologies for gene delivery and genome editing with modern techniques for insertion of the neurotrophic factors into clinically relevant cells for a more sustained pharmaceutical effect. It has been suggested that the long-term expression of neurotrophic factors is the ultimate approach to prevent and/or treat neurodegenerative disorders such as glaucoma in patients who do not respond to available treatments or are at the progressive stage of the disease. Recent preclinical research suggests that novel neuroprotective gene and cell therapeutics could be promising approaches for both non-invasive neuroprotection and regenerative functions in the eye. Several progenitor and retinal cell types have been investigated as potential candidates for glaucoma neurotrophin therapy either as targets for gene therapy, options for cell replacement therapy, or as vehicles for gene delivery. Therefore, in parallel with deeper understanding of the specific protective effects of different neurotrophic factors and the potential therapeutic cell candidates for glaucoma neuroprotection, the development of non-invasive and highly specific gene delivery methods with safe and effective technologies to modify cell candidates for life-long neuroprotection in the eye is essential before investing in this field.

## Introduction

Glaucoma is one of the most common causes of blindness in the world with over 70 million people (79 million by 2020), including 400,000 Canadians, affected by this disease (Foster and Resnikoff, 2005). While preventable with proper diagnosis and continual treatment, a patient's vision cannot be recovered once it has been affected. The underlying mechanisms associated with glaucoma progression are still under investigation, but it is well established that the damage to retinal ganglion cells (RGCs) is mainly the result of mechanical injury resulting from increased intraocular pressure (IOP) caused by disruption of the trabecular meshwork (Margalit and Sadda, 2003).

Local vascular insufficiency at the optic nerve head can also lead to a decrease in neurotrophic factors (NFs) levels (Bessero and Clarke, 2010), which results in RGC death (Fang et al., 2010). Currently, glaucoma management relies on pharmacological and invasive surgical treatments mainly by reducing the IOP, the most important risk factor for the progression of the visual field loss.

There is strong evidences from several research groups that repeated administration of neurotrophic factors (NTFs) such as neurotrophin-4 (NT-4), brain-derived neurotrophic factor (BDNF) (Di Polo et al., 1998), ciliary neurotrophic factor (CNTF) (Ji et al., 2004), and glial cell line-derived neurotrophic factor (GDNF) (Jiang et al., 2007) increased the survival of neurons in rodent models. Among the NTFs, BDNF appears to provide the highest level of protection by supporting both protective and regenerative functions (Danesh-Meyer, 2011). BDNF has a direct effect on RGCs and appears to correct problems with bidirectional transport of NTFs and by indirect influence on other retinal cells, helps direct damaged axons. Initial damage to axonal transport has already been attributed to a deficiency in NTFs. For instance, neurotrophin deprivation due to bidirectional axonal transport obstruction within RGCs has been shown to result in axonal damage (Iwabe et al., 2007). Retrograde flow at the optic nerve head prevents proteins made by RGCs from reaching their axonal extensions and perturbed retrograde transport of NTFs produced in the superior colliculus (SC) in the brain to reach the RGCs (Lim et al., 2010). Several studies have now identified the potential role of astrocytes, microglia and Müller cells in RGC survival within the optic nerve head region through their secretion of NTFs (Johnson and Morrison, 2009). This has been suggested by studies involving adenovirus-mediated intravitreal delivery targeted toward Müller cells (Di Polo et al., 1998) and after a single intravitreal injection of BDNF in a cat model (Weber et al., 2008). The targeting of regenerative factors to Müller glial cells can also stimulate their dedifferentiation into multipotent progenitor cells, which may differentiate into new RGCs or photoreceptor cells to replace the ones that became damaged during injury by high IOP. BDNF also appears to support axonal path finding to the brain (Benowitz and Yin, 2008, 2010).

It is now widely recognized that lowering IOP in the treatment of glaucoma is not enough. In addition to neuroprotective and neuroregenerative approaches, poly-therapeutic strategies may be the future. Combination treatments such as IOP-lowering drugs with neurotrophic factors and/or antioxidants and/or anti-apoptotic agents may be necessary. The common challenge to all these therapeutic possibilities lies in the delivery and maintenance of NTF levels in the retina for a prolonged period.

Neurotrophic factor-based gene therapy may meet this challenge. It could be performed either by direct transfer of transgenes coding for NTFs into the patient or by using living cells that express NTFs persistently as vehicles to transport the NTF transgenes. In general, gene delivery and prolonged or stable expression of the intended therapeutic transgenes in target cells are the key factors to a successful gene therapy approach.

In part I of this paper we discussed the supportive effects of different NTFs in glaucoma, identified different methods of NTF transgene delivery, and discussed potential cell candidates for cell-mediated therapy. Here, we focus the review on advanced techniques for production of safer and more efficient DNA vectors as well as innovative non-viral approaches for *ex vivo* gene delivery/gene editing in order to provide stable and long-term expression of therapeutic genes such as NTFs in suitable candidate cells.

## RGC rescue therapy in glaucoma treatment

Exogenous supplementation of NTFs, apoptosis inhibitors and survival factors as transgenes or their recombinant protein products is a promising approach to stop or decline RGC death in progressive glaucoma (Thumann, 2012).

Interrupting the apoptosis cascade by delivering genes encoding caspase inhibitors or expressing anti-apoptotic genes such as *Bcl-2*, or interfering with the expression and activity of pro-apoptotic factors by siRNA technology have been successfully tested in preclinical studies of glaucoma treatment in animal models (Liu et al., 2009; Thumann, 2012).

Additionally, increasing NTF levels by gene delivery has been widely investigated *in vitro* and *in vivo*. NTFs are small proteins that are secreted by the central and peripheral nervous system and are critical in their own development and maintenance (Lim et al., 2010). NTFs are classified into several groups and among them, the nerve growth factor (NGF) family members such as GDNF, BDNF, NTs, and CNTF have been the subject of more detailed studies for gene therapy in glaucoma(Johnson et al., 2011).

NTF supply is important for RGC survival or regeneration during development, and extensive experimental strategies have been tested to supply exogenous NTF to protect and promote survival of injured RGCs in glaucoma.

Although direct gene therapy by delivering the exogenous transgenes encoding for NTFs using different viral and non-viral carriers are particularly attractive, in certain cases such as glaucoma, life-long neuroprotective support through exogenous NTF therapy is essential. Therefore, cell therapy would be a more sustainable approach *via* delivering NTFs by living cells and direct replacement of growth factors and NTFs by cells that are genetically modified *ex vivo*. Application of genetically modified cells as gene delivery vehicles has certain advantages such as relative simplicity of manipulation and evaluation of cells *in vitro* compared to *in vivo* gene modification. Furthermore, some of these modified cells continue to divide *in vitro* under certain culture conditions, which facilitates expansion of these cells for further investigations. Finally, some of these engineered cells show a tendency to localize into particular tissues.

Recent studies showed that several stem and progenitor cells expressing and secreting the NTFs provide neuroprotective support when transplanted into animal models of glaucoma and other retinal diseases (Johnson et al., 2011). In this paper we focus on advanced non-viral nanotechnology tools for genetic modification of candidate cells aiming to achieve long-term expression of NTFs therapeutics.

## New generation of DNA therapeutics

**Figure 1:**
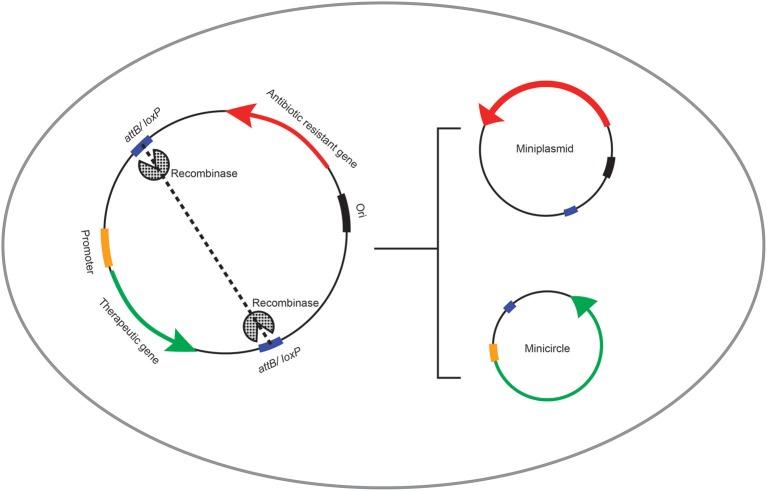
**Schematic representation of the recombinase-mediated production if minicircle DNA vectors**. *In vivo* production of bacterial sequence-depleted DNA vectors with phage-derived enzymes is processed *via* insertion of the recognition sequence (*attB* or *loxP* site) of recombinase enzymes into the intended plasmid DNA vector closely upstream and downstream of the therapeutic gene expression cassette. This plasmid is then transferred into the engineered *E.coli* cell capable of expressing the recombinase enzyme. Consequently, the *in vivo* intramolecular recombination at the recognition sites results in generation of two smaller molecules from the single parent plasmid DNA: (i) the minicircle DNA vector comprising the therapeutic gene expression cargo, and (ii) the miniplasmid containing the bacterial sequences including the origin of replication and the antibiotic resistant gene.

The necessity to generate safe and efficient DNA vectors for transgene delivery *via* a variety of non-viral approaches has spurred many different proposals. Among them bacterial sequence free DNA vectors in two forms such as supercoiled circular covalently closed and linear covalently closed DNA, termed as “minicircle” and “ministring,” respectively, are considered the most promising (Darquet et al., 1997, 1999; Chen et al., 2003; Nafissi and Slavcev, 2012; Nafissi et al., 2014; Slavcev et al., 2014; Slavcev and Nafissi, 2014). Replication and largescale production of plasmid DNA vectors is dependent on the prokaryotic backbone and specific selection markers to isolate and propagate plasmid-containing bacterial strains after bacterial transformation. However, these sequences are undesirable in clinical applications because of the following reasons: (A) the bacterial sequences are recognized as invading factors and trigger host innate immune response that leads to systematic removal of the vector (Klinman et al., 1996; Mitsui et al., 2009); (B) the horizontal transfer (importing genes from environment or from other bacteria) of antibiotic resistant genes from plasmid DNA to normal microbial flora is a risk factor for the generation of antibiotic resistant flora (Chen et al., 2008); (C) residual selection markers in the final plasmid product, due to unsuccessful removal, can cause allergic reaction and hypersensitivity in sensitive individuals after gene delivery (Cavagnaro, 2013); and (D) the bacterial sequences are reported as the main cause for heterochromatin-dependent silencing of the intended transgene (Chen et al., 2003; Mayrhofer et al., 2009). In contrast, the new generation of DNA vectors that are bacterial sequence free offer higher and more persistent expression, generally at levels 100–1000 times greater than their standard plasmid precursor (Kay, 2011). Previously, purification of miniDNA vectors from bacterial extracts was labor-intensive, time-consuming, and a multi-step process that needed digestion of the bacterial backbone by a restriction enzyme (Schakowski et al., 2001, 2007) followed by purification of miniDNA vector and removal of digested sequences by cesium chloride ultracentrifugation (Bigger, 2001; Chen et al., 2005). However, employing prokaryotic-derived site-specific recombination systems, mainly from bacterial viruses (phage) such as λ integrase (Int), P1-derived, Cre, phiC31Int, N15-derived TelN, and PY54-derived Tel, dramatically facilitated the production and purification of miniDNA vectors (Nafissi and Slavcev, 2014). These systems show limitations that have been improved over the time (Table 1). In general, the first step in generating bacterial sequence-depleted DNA vectors with phage-derived enzymes is to engineer a bacterial cell (mainly *E.coli*) that express these enzymes, insert the recognition sequence of these enzymes in to the plasmid DNA vector closely upstream and downstream of the therapeutic transgene expression cassette, and transfer the plasmid into engineered *E.coli* cell (Nafissi and Slavcev, 2012). Consequently, the *in vivo* intramolecular recombination at the recognition sites results in generation of two well-characterized molecules from the single parent plasmid:(i) the miniDNA vector (circular or linear covalently closed) comprising the therapeutic transgene expression cassette, and (ii) the miniplasmid containing the bacterial backbone elements (Figure 1). Combining the endonuclease I-*Sce*I together with its recognition site in the plasmid backbone allows simultaneous digestion of the bacterial backbone into small pieces and production of purified miniDNA vector. For instance, further purification of the minicircles by affinity-based chromatography allows the isolation of highly pure and pharmaceutical-grade minicircles by this technique (Rodríguez, 2004; Thyagarajan et al., 2008). For the first time, Darquet et al. (1997, 1999) showed that “minicircle” DNA confers much higher transgene expression levels *in vitro* and *in vivo*, respectively, compared to the parental plasmid precursors or other conventional control plasmids encoding the same transgene. This result was further confirmed, and showed an even more significant increase in the expression of the encoded transgene, when the same amount (weight-to-weight basis) of minicircle DNA and parental plasmids were delivered (Darquet et al., 1997, 1999; Chen et al., 2003, 2005; Vaysse et al., 2006; Jia et al., 2010). After developing the convenient production systems mentioned above, miniDNA vectors have been extensively studied by different research groups by comparative studies with the parent plasmids (Table 2). The following examples reflect the broad applicability of the new generation of DNA vectors from gene therapy to more recently in stem cell research and regenerative medicine considering dynamic aspects from formulations and optimization of miniDNA vectors complexes with synthetic vectors. Neural stem cells (NSC) that are very difficult to transfect were successfully transfected by minicircle DNA vector by microporation and showed higher transgene expression and NSC survival when compared to their plasmid counterpart (Madeira et al., 2013).

**Table 1:** ***In vivo* phage-derived systems for production of minicircle DNA vectors**.

**Phage-derived production systems**	**Advantages**	**Limitations**	**Pharmaceutical scaled production**
Phage λ *attB*-*attP* recombination		Size exclusion/sequence-specific purification; low recovery due to bidirectional recombination reaction(Nafissi and Slavcev, 2014)	Not possible
FLP/frt site-specific recombination		Bidirectional and fully reversible recombination reaction, results in generation of multimeric DNA structures (Sadowski, 1986)	Not possible
Cre/mutated loxP site-specific recombination	Unidirectional recombination reaction, tight control of recombinase gene expression, generating of monomeric minicircle molecules (Bigger, 2001)	Size exclusion/sequence-specific purification, co purification of bacterial sequence is an issue	Not possible
ΦC31 integrase	Unidirectional recombination reaction, tight control of recombinase gene expression, generation of monomeric minicircle molecules, *in vivo* enzymatic degredation of bacterial sequence results in facilitated purification, high-yield production of minicircles (Kobelt et al., 2013)	Not reported	Possible

**Table 2:** **Features of miniDNA vectors compared to standard plasmid DNA vectors**.

**General features**	**Plasmid DNA**	MiniDNA^*^
	Replicative in bacterial host	Non-replicative in bacterial host
	Supercoiled	Supercoiled or circular/linear closed
	Bacterial origin of replication	No bacterial origin of replication
	Bacterial selection marker (Antibiotic resistance gene)	No bacterial selection marker (Antibiotic resistance gene)
	Relatively high immunostimulatory unmethylated CpG dinucleotides^**^	No to very few immunostimulatory unmethylated CpG dinucleotides
	Large-scaled production is available	Large-scaled production is available
	Pharmaceutical-grade purification is available	Pharmaceutical-grade purification is available
	Medium to large size^***^	Relatively smaller than plasmids

* *Bacterial sequence depleted*.

** *Depends to the size*.

*** *Depends to the size of therapeutic transgene*.

Minicircle DNA vectors have been also used as integrating vectors for recombinase-mediated cassette exchange (RMCE) (Jakobsen et al., 2013) or as a sleeping beauty transposition system (Sharma et al., 2013), and minicircles significantly improved the integration efficacy and gene modification capacity, respectively, compared to the standard plasmids. In a different study, same molar ratio and same copy numbers of the minicircle and plasmid DNA vectors were complexed with lipid-based nanoparticles and were transfected into a variety of human cell lines and in animal models. This study confirmed previous results and showed that transgene delivery by minicircle DNA vectors significantly improves transgene expression levels both *in vitro* and *in vivo*. This group also concluded that improved minicircle delivery *in vivo* and higher transgene expression levels are due to the compact size of minicircle-nanoparticles, better cellular uptake and cell entry, higher intercellular minicircle copy numbers in transfected cells, better intracellular trafficking toward nuclei, and better nuclear uptake. These results also showed higher mRNA transcription levels in minicircle-mediated transgene delivery compared to parent plasmid-mediated transgene delivery (Kobelt et al., 2013). They concluded that the euchromatin structure and more accessibility of bacterial-sequence-free DNA vectors to transcription machinery of host cell is a key reason for higher expression level of transgene. Even further modification of miniDNA vectors to combat intracellular barriers such as nuclear membrane would dramatically improve transgene delivery, especially in slow or non-dividing cells. For example it was shown that the addition of nuclear-targeting sequences (DTS), such as the SV40 enhancer, or karyopherins (Miller and Dean, 2009) to the DNA sequence in parallel with removing undesired bacterial sequences improved transfection efficacy and expression levels of the transgene and its protein product (Nafissi et al., 2014).

New generation of DNA vectors represent a promising alternative to conventional plasmids in terms of biosafety, bio- and immuno-compatibility, improved gene transfer, potential bioavailability and cytoplasmic diffusion due to their smaller size (Nafissi et al., 2014), and low immunogenicity due to the lack of bacterial sequences and immunogenic motifs (Chen et al., 2003; Vaysse et al., 2006). Although miniDNA vectors provide more sustained expression of the transgene relative to standard plasmid DNA vector, in more rapidly dividing cells and tissues with higher regenerative capacity, most DNA vectors become diluted after each mitosis and eventually disappear. Therefore, permanent introduction of a therapeutic transgene followed by its sustained expression is more desirable where life-long pharmaceutical effect is needed. In particular, in the case of gene or cell mediated therapy of glaucoma and other neurodegenerative disorders that require life-long and stable expression of NTFs, tightly controlled integrating DNA vectors and strategic genetic engineering systems are required (Jandial et al., 2008; Blurton-Jones et al., 2014). Therefore, better and safer techniques of genome editing could open a new avenue to investigate and treat neurodegenerative disorders more effectively.

Permanent expression of therapeutic genes is generally carried out by three different approaches: (i) random integration (illegitimate insertion); (ii) homologous recombination (HR); or (iii) site-specific insertion of the transgene into the chromosome of an anticipated cell. Random integration is mostly carried out by viral vectors to achieve insertion of an exogenous transgene into the host human cell's genome. However, lack of control over the site and position of integration would result in undesirable side effects such as unpredictable expression level or silencing of the integrated transgene, potential mutagenesis of neighboring genes, activating oncogenes/deactivating tumor suppressor genes, and eventually cancer. Therefore, random integration by viral vectors is not a safe method for permanent and sustained expression of therapeutic transgene in *ex vivo* cell-mediated therapies. HR and site-specific insertion are indeed the methods of choice for the optimal control over the site of transgene integration, the number of copies inserted per target cell, the expression level of therapeutic transgene, and to reduce the risk of oncogenesis. Safe and efficient site-specific insertion is carried out through bacteriophage integrase-mediated and transposon systems, and HR through recent and efficient techniques involving Zinc Finger Nucleases (ZFN), Transcription Activator-Like Effector Nucleases (TALENs), and Clustered Regularly Interspaced Short Palindromic Repeats (CRISPR).

## Site-specific recombination for cell therapy

The interest in precise gene modification is growing dramatically owing to recent advances in targeted genome engineering. Targeted genetic engineering techniques allow very specific modifications such as gene insertion, deletion, and replacement. Permanent modification of cells has been extensively used in regenerative medicine as a new clinical tool.

Some examples are engineering glial progenitor cells to permanently express adhesion molecules to increase homing of these cells into the brain (Gorelik et al., 2012), or enhancing the therapeutic effects of stem cells by permanent insertion of NTF genes (Crigler et al., 2006; Janowski and Date, 2011). Here we briefly review some of the most popular and safe non-viral methods for genetic engineering of clinically relevant cells and insertion of therapeutic transgenes such as NTF encoding genes into anticipated cells by transposon systems and bacteriophage-mediated integrase systems.

## Transposon systems

Non-viral transposon systems have been widely used to generate genetically modified and clinically relevant human cells including but not limited to induced pluripotent stem (iPSC), embryonic stem cells (ESC), and mesenchymal and hematopoietic stem cells (Saha et al., 2015). “*Sleeping Beauty* transposon system (SBTS)” and “*piggyback (PB)* transposon system” are the two transposon systems that have been successfully used as non-viral gene delivery carriers for gene modification and generation of clinical grade human cells for gene and cell therapy. These systems have lower cost for design, construction, and production of pharmaceutical grade products on a large scale as well as stimulating low levels of innate immunity and the capacity to co-deliver multiple therapeutic transgenes compared to viral integrating systems.

## *Sleeping Beauty* transposon system (SBTS)

“*Sleeping Beauty* transposon system (SBTS)” is a non-viral plasmid-based integration system that combines the integration benefits of viral vectors and ease of production and manipulation of naked DNA vectors and ease of delivery by nanoparticles. The SBTS consists of (A) a plasmid transposon carrying the therapeutic transgene expression cassette flanked by the terminal Inverted Repeats (IR) that contain binding sites for the transposase enzyme; and (B) a source of transposase enzyme, which is a 360-amino acid DNA-binding protein with a transposon binding domain, a nuclear localization signal (NLS), and a catalytic domain.

**Figure 2:**
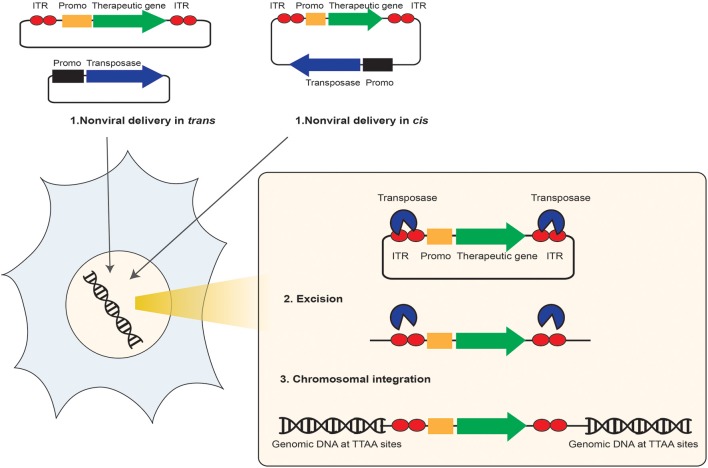
**Schematic representation of the non-viral transposon-mediated site-specific gene therapy**. The non-viral transposon is used as a bi-component DNA vector system for delivery and insertion of therapeutic transgenes from a DNA vector into the target cell chromosome. This system is composed of either one plasmid (cis) or two plasmids (trans) contains the therapeutic gene of interest (GOI) (green arrow) and its expression cargo such as promoter (orange box) flanked between the two transposon Inverted Terminal Repeats (ITR) (red circles), and the transposase enzyme (blue arrow) expression cargo. (1) After delivery into the intended target cell and transportation into the nucleus, through a “cut and paste” transposase activity, (2) the transposon is excised from the transposon donor DNA vector, and (3) the therapeutic transgene is integrated at a chromosomal site.

The SBTS systems mostly target the TA rich sites of the hosting genome to insert the intended therapeutic transgene expression cargo. The gene encoding for the transposase enzyme can be assembled either on the same plasmid that express the therapeutic transgene (sis) or can be located at a different plasmid (trans). In case of trans expression, the two DNA vectors (one carries the transposase enzyme encoding gene and the other carries the therapeutic transgene expression cassette) could be packed within the same nanoparticle carrier and co-transferred into the intended host cell. Few hours after delivery, the transposon system would reach the nucleus of the host cell, the transposase enzyme would be expressed in the cytoplasm and the NLS that has been added to its sequence would direct import of the transposase into the nucleus, cut the IR sequences, and facilitate relocation and insertion of the therapeutic transgene expression cargo from DNA vector into the host genome (Aronovich et al., 2011) (Figure 2). Similar to other technologies, SBTS system needs to be improved before entering into clinical trials. For example, in order to achieve pharmacologically relevant expression level of the therapeutic gene and just sufficient transposase expression to carry out the cut-and-paste reaction in difficult-to-transfect target cells, better delivery techniques are required. In addition, precise understanding of the target cell genome would assure insertion of the therapeutic gene into the TA sites and avoids the side effects of insertion into undesired loci.

Transposase expression level and duration of expression is critical in transposon systems because very high expression level of the transposase would cause insertion of the transgene into many different locations of the hosting genome that are rich in TA. In addition, long-term expression of the transposase is also not desirable because it might cause the excision of the integrated transgene out of genome and reverse the effect. Therefore, a good understanding of the host's genetic background, type of tissue to be targeted, and therapeutic gene vs. transposase expression level is necessary to improve transposition efficacy (Aronovich et al., 2011). The CMV promoter would provide a high but short-term expression of the SB transposase enzyme in many human cells and therefore would inhibit the re-mobilization of the integrated therapeutic gene after a longer time (Hackett et al., 2011). On the other hand, tissue-specific mammalian promoters derive an alternative promoter for the therapeutic transgene for sustained expression of the transgene in the right site (Kachi et al., 2006). The SB100X “plasmid-based integrating” transposon system in combination with electroporation technology (Mátés et al., 2009) was recently applied to permanently knock-in the anti-angiogenic and neuroprotective factor PEDF into the ARPE-19 human retinal pigment epithelial cells under the control of either CMV or cell specific CAGGS promoters. In this study, the PEDF positive cells were successfully transplanted into the retina to treat age-related macular degeneration (AMD) (Johnen et al., 2012).

As the proof of principle for licensed SBTS technology in clinical trials it is worth to point out few examples, even though these trials are not specifically toward the treatment of glaucoma. (1) In a in phase II clinical trial of cancer immunotherapy research, patients-derived T cells were genetically modified by SBTS system to express a chimeric antigen receptor (CAR) and were injected back to patients with B-cell malignancies in order to redirect the specificity of human T cells in these patients (Hackett et al., 2011). (2) SBTS technology provided life-long expression of Fanc-C and Fanc-A genes to treat Fanconi anemia, or Factor VIII gene to treat Hemophilia A (Nienhuis, 2008). (3) SBTS-mediated life-long expression of IL13-HSVTK was achieved to treat brain tumors (Di Matteo et al., 2012).

In the field of stem cell research, plasmid-based SB systems have been used in combination with nanoparticles for the expression of therapeutic genes. For example, four human iPSC lines were successfully generated and characterized from fetal fibroblasts using SB-based DNA vector that were combined and delivered by nanocarriers (Davis et al., 2013). Also, rat mesenchymal stem cells (rMSCs) were efficiently transfected by a nanoparticle called “liposome protamine/DNA lipoplex (LPD).” LPD was electrostatically assembled from cationic liposomes and an anionic complex of protamine, SB transposon system, and NLS targeting peptides in order to enhance cell specific targeting and nuclear uptake. This complex dramatically improved transposon-mediated gene insertion in MSCs (Ma et al., 2013). As such, the non-viral DNA-SB-based therapeutics in combination with engineered cell specific nanoparticles serve as a promising and long-lasting way to insert therapeutic genes into the chromosomes of host cells without using a viral integration systems and their consequent undesirable side effects (Aronovich et al., 2011).

## *PiggyBac* transposon system

The *PiggyBac (PB)* transposon derived originally from Lepidopterans is composed of two identical short inverted terminal repeats (700 bp) and a transposase-encoding sequence (coding a 594 amino-acid transposase). The *PB* transposase catalyzes the transposition of the therapeutic gene expression cargo that is flanked by the inverted terminal repeats carrying by a plasmid and insertion of therapeutic transgene into TTAA rich sites of the host genome. The short IR are the key elements of the PB transposition system (Figure 2). One of the benefits of *PB*transposon system over SBTS is the capacity of this system to deliver larger transgenes, complex genes, or multiple therapeutic genes together with their regulatory regions. However, low transfection efficiency of large plasmids is still the major limitation of *PB*transposon system (Kim et al., 2011). In terms of safety, one of the concerns about using PB systems in clinical applications in human cells is the distribution of over 2000 PB-like elements in the human genome which raises the risk of genome rearrangement and the potential for remobilization of the integrated transgene by endogenous transposase expression. However, recently the potential undesired PB-mediated genomic rearrangements have been investigated to determine the safety of PB systems in clinical applications. No genomic rearrangement and re-mobilization of the integrated transgene were observed, but it was also suggested that long-term evaluation of the safety of transposase systems in animal models is needed to reach a final conclusion (Saha et al., 2015). Therefore, despite these observations, insertional mutagenesis in unwanted sites or rearrangement of the neighboring genes and gene remobilization after integration of the therapeutic gene into host genome still remains the major limitation of PB transposon system in the precise modification of clinically relevant stem/progenitor cells (Li et al., 2013). The PB system was efficiently applied to reprogram murine and human embryonic fibroblasts and to generate pluripotent stem cells. As such, four transcription factors (c-Myc, Klf4, Oct4, and Sox2) were inserted into the genome of embryonic fibroblast cells to reprogram them and to generate pluripotent stem cells. Furthermore, applying the natural tendency of PB transposase to excise the inserted transposon, transcription factors were removed from well-established iPSC cell lines (Woltjen, 2009) post reprogramming. The PB system was also successfully applied in field of cancer immunotherapy by isolating T cells from patients with malignancies and generating genetically modified tumor-antigen-specific T cells. These cells were further injected to patients and the anti-tumor activity of modified T cells with one or multiple insertions was evaluated (Nakazawa et al., 2009). Several studies were carried out using the PB transposon plasmid DNA vectors combined with polymer- or lipid-based nanoparticles to enhance transgene delivery and insertion of the intended gene through PB transposase activity in mammalian cells (Palavesam et al., 2013; Chakraborty et al., 2014).

The miniPB system was recently generated and applied to insert the GOI into the host genome with the same integration efficiency but using a much smaller plasmid vector with the advantage of enhanced transfection efficiency *in vitro* and *in vivo* (Solodushko et al., 2014). The mini PB plasmid is a single-plasmid system (cis) carrying the transposase gene and very short inverted terminal repeats (35 bp) flanking the therapeutic transgene expression cassette (Yusa et al., 2009; Li et al., 2011).

Compared to the PB transposon system, the SB100X shows superior efficacy in most human cells including hematopoietic cells with lower safety risk of random integration and insertional mutagenesis (Aronovich et al., 2011). However, for multiple therapeutic gene insertion, the PB system remains the method of choice (Saha et al., 2015).

### ΦC31 integrase

**Figure 3:**
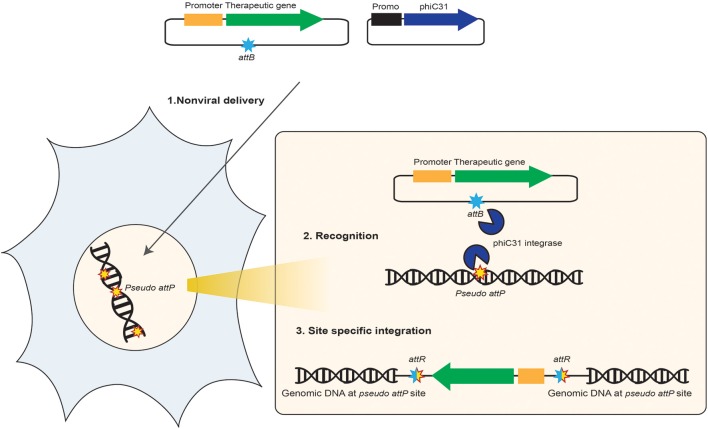
**Schematic representation of the non-viral integrase-mediated site-specific gene therapy**. This diagram shows how ΦC31 integrase system benefits in gene therapy. (1) One DNA vector that carries the desired therapeutic transgene expression cargo (promoter, transgene, reporter gene, polyA signal) and also the *attB* site that is recognized by ΦC31 integrase enzyme and one DNA vector that carries the integrase encoding gene are co-delivered to human target cells. Both DNA vectors enter nucleus, the integrase is produced in the cytoplasm and redirected to the nucleus. (2) ΦC31 integrase enzyme binds as a dimer to the *attB* site on the therapeutic DNA vector and bounds at pseudo *attP* sites present in the human genome. (3) A cut-and-paste recombination reaction occurs at the site of integration, which results in the insertion of the therapeutic transgene expression cargo into the chromosome of human cells at the pseudo *attP* site. This process provides long-term expression of the transgene and production of therapeutic protein in the desired target cells.

Site-specific DNA insertion systems are derived from prokaryotes and unicellular yeasts and a number of them have been exploited to facilitate efficient DNA exchange in human cells (Nafissi and Slavcev, 2014). In fact, all site-specific integration systems typically mediate efficient “cut-and-paste” type of DNA exchange between recognition sites in the range of 30–40 bp or longer. Invading bacterial viruses (phage) use this system to integrate their genome into their bacterial host chromosomes by a reaction catalyzed by integrase enzymes at short sequences, termed as the “phage attachment site (*attP*)” and “bacterial attachment site (*attB*)” (Nafissi and Slavcev, 2014). Calos and her colleagues showed that sequences very similar to wild type *attP* and *attB* sites are available in the human genome and called them as “pseudo *attP*” and “pseudo *attB*,” sites because an exogenous gene can be integrated specifically into these sites in presence of a phage integrase (Chalberg et al., 2006). The actinophage ΦC31 integrase, discovered in the 1990s, efficiently catalyzes a site-specific genomic integration between two DNA recognition (Groth and Calos, 2004) sequences: *attB* containing DNA vector and a pseudo *attP* site within the genome of an anticipated human cell, leading to permanent transgene expression (Chavez et al., 2011). Using this system, the insertion of a foreign DNA such as a therapeutic transgene is characterized by the following events: (1) recombination occurs at a specific site on the interacting DNA molecules: DNA vector and hosting cell genomic DNA; (2) expression and synthesis of the recombinase enzyme in the host cytoplasm using host protein synthesis machinery; (3) re-location of the integrase to the host cell nuclei using nuclear localization signals; (4) strand exchange occurs at small regions of DNA homology within the recognition sites; (5) pairing of the interacting insertion sites followed by strand exchange results in structural intermediates; and (6) resolution of intermediates followed by strand migration (Groth et al., 2000) (Figure 3). In the human genome “pseudo *attP*” sites show 40% similarity to wild-type *attP* sequence. In comparison with the “TA” and “TTAA” rich sequences that are the target sites for the *SB* and PB transposon systems, respectively, pseudo *attP* are mostly located in transcriptionally active, euchoromatin sites, and exons with less frequency of introns. ΦC31 integrase provides one-copy integration of the exogenous transgene per cell without disrupting the endogenous genes. Therefore, integrase-mediated site-specific gene insertion of transcription factors offers an alternate method of producing iPS cells without interfering with endogenous gene functions (Lan et al., 2012). ΦC31 integrase-mediated insertion of exogenous gene occurs at 10-fold higher rates than random integration and often provides higher expression levels than those inserted randomly into the human genome that possibly causes either silencing, undesired side effects of over expression of the therapeutic gene, or oncogenesis/genotoxicity (Nafissi and Slavcev, 2014). In addition, bacteriophage ΦC31integrase catalyzes unidirectional integration of therapeutic gene into only pseudo *attP* sites in the human genome and eliminates the risk of excision of inserted transgene (Chalberg et al., 2006), which is the most important advantage of this system over transposon systems. Transgenic animals generated by ΦC31integrase-mediated site-specific insertion never showed any cancer development (Calos, 2006), or the human ESC that been genetically modified by ΦC31 integrase retained their ability to differentiate normally into all three germ layers (Thyagarajan et al., 2008). In animal models, for example, hereditary tyrosinemia type I and muscular dystrophy was treated by ΦC31 integrase–mediated integration of fumarylacetoacetate hydrolase and dystrophin, respectively. In these studies, plasmid DNA carrying the *attB* site and the therapeutic transgene were co-delivered with plasmid DNA expressing integrase by hydrodynamic tail vein injection (Held et al., 2005) or intramuscular injection followed by electroporation (Bertoni et al., 2006).

A comprehensive study confirmed the biosafety of ΦC31 integrase-mediated integration of therapeutic transgene into human umbilical cord lining epithelial cells (CLECs) as source for several stem-cell like cell types (Sivalingam et al., 2010).

Any plasmid DNA-based integration system can be combined with synthetic carriers to form nanoparticles that are very efficient in transfecting human cells and transferring the DNA cargo into the nuclei. Different studies were carried out to improve this system. Very recently, Oliveira and colleagues combined polymer-based nanoparticles with integrase technology to create ΦC31-chitosan-mediated gene delivery and integration system. They have characterized the nanoparticles and significantly improved the transfection and integration efficacy in human embryonic kidney cells. They achieved higher expression levels of small and large integrated transgenes for over 10 weeks post transfection (Oliveira et al., 2015). Ease of transfection into mammalian cells using reliable synthetic formulations and variety of nanoparticles makes the DNA-based integrating systems superior compared to their viral counterparts, in particular in terms of immunocompatibility and safety with genotoxicity perspective.

## Homologous recombination for cell therapy

**Figure 4:**
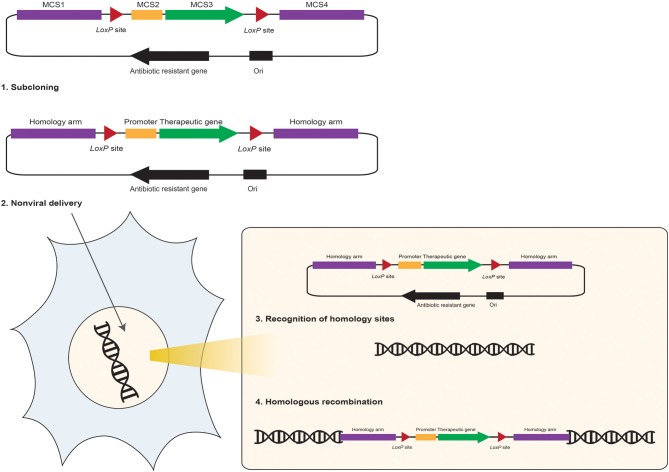
**Schematic representation of the DNA vector-mediated homologous recombination in mammalian cells**. The homologous recombination donor plasmid DNA vector carries four multiple cloning sites (MCS) to facilitate: (1) sub-cloning and insertion of a therapeutic gene (sub-cloned at MCS3) and it's promoter (sub-cloned at MCS2) to a desired site of the host genome *via* two homology arms (sequences homolog to the insertion site are sub-cloned at the MCS1 and MCS4 flanking the therapeutic gene expression cargo). (2) Non-viral delivery of the HR donor DNA vector into the anticipated cell provides translocation of this vector into the nuclei. (3) Homology arms facilitate recognition of homolog sequence on the target cell's genome. (4) HR occurs at the homology sites and the therapeutic transgene expression cargo would integrate into the host chromosome through HR. Adding the *LoxP* sites (red triangles) to the HR donor DNA vector would facilitate removal of homology arms, or other unnecessary sequences, after insertion of the therapeutic cassette into the host genome *via* expression of the Cre recombinase and its excision activity on the *loxP* sites through “Cre/LoxP recombination systems.”

Unlike viral vectors that usually integrate into the host chromosome in an uncontrolled fashion, one of the most policed methods of genome modification is to insert the therapeutic transgene into the recognized and desired location by homologous recombination (Figure 4). HR was defined as critical process involved in genetic diversity and repair of DNA double-strand breaks (DSBs) in pro- and eukaryotic cells (Kakarougkas and Jeggo, 2014). The process of HR is very important in maintaining the chromosome integrity and to protect and recover any open ended DNA caused by environmental assaults or invading DNA. This process involves the alignment of similar DNA sequences to form a mobile junction between four strands of DNA, termed “Holiday junctions,” which are highly conserved in both prokaryotic and eukaryotic cells. HR needs energy and cofactors from host cells (Nafissi and Slavcev, 2014) and the presence of DNA DSB in mammalian cells is the essential step to stimulate HR (West, 2003). The frequency of integration by HR appears to be generally low, about 10^−6^, for most mammalian cells, but it was reported that this can be increased up to 100 fold by making DSBs at the target site by restriction enzymatic reactions such as CRISPRs, TALENs, and ZFNs. Some of the drawbacks of HR system is the general low frequency of HR in mammalian cells, providing the homology arms in the donor DNA vector carrying therapeutic gene, and the resources of enzymes to generate DSB in hosting target sequences (Pan et al., 2013; Yin et al., 2014b). Their sequence-specific binding modules can recognize unique nucleotides in the genome along with the fused endonuclease such as FolK1, Cas9, or ZFN to specifically induce a double-stranded break (DSB) in the chromosome. All endonuclease systems introduce DSBs in the target DNA and lead to recruitment of the cellular repair machinery, which can drive HR with dramatically higher frequency at the cleavage site in the presence of complementary sequence arms that are cloned into HR donor plasmid DNA vectors. These systems have been applied in repair of damaged DNA, gene disruption, gene insertion, gene correction and point mutagenesis, and chromosomal rearrangements (Doudna and Charpentier, 2014; Kim and Kim, 2014). Advanced genome engineering tools are now commercially available. For example, the TALENs, ZFNs, and CRISPR technologies are available from Addgene, Sigma-Aldrich, and Life Technologies.

## Zinc finger nucleases

As it was mentioned, the process of HR in human cells is indeed too inefficient to lead to a reliable therapeutic result and creating DNA breaks is the key step to increase efficacy of this process. Therefore, ZFNs have been widely used to create the gene-specific DNA breaksand enhanced the HR efficacy to several orders of magnitude (Porteus, 2006, 2008). Zinc Finger DNA Binding Proteins (ZFPs) are a class of DNA binding proteins that naturally exist in prokaryotes. Through modification of the DNA recognition and binding function of ZFPs, it is possible to direct ZFPs to a target sequence of DNA, which enables the site-specific localization of the ZFPs in a designated sequence. ZFPs have been developed to either regulate gene expression by a turning on and off mechanism, or correct genes using endonuclease enzymes (Davis and Stokoe, 2010). ZFNs are artificial proteins composed of a DNA-binding protein and a nuclease protein that is fused to the first protein. Two ZFNs protein domains are required to bind to DNA, to dimerize and activate the nuclease domain, and to create a DSB (Porteus and Baltimore, 2003). A ZFN subunit encompasses three to six zinc-fingers arranged in a tandem repeat and a catalytic domain of an endonuclease enzyme, like FolK1, in such a way that a short linker connects the two domains. At the ZFN target site located at the host genome, the two ZFN subunits are dimerized and the nuclease is activated and cuts the DNA. Precise ZFN-mediated gene targeting involves several steps as follows: (1) identify the full ZFN-binding site within the target GOI; (2) design a pair of ZFNs; (3) test the ZFN pair for activity; (4) identify a targeting construct to create the desired genomic modification; (5) co-transfer the ZFNs and the targeting vector to create the breaks and to insert the therapeutic transgene at the target site, respectively (Jamieson et al., 2003). ZFN derived HR was applied in different clinically relevant studies. In the field of neuroscience, engineered ZF protein transcription factors (ZFP TFs) has been used to improve expression of endogenous glial cell-derived neurotrophic factor (GDNF) in a rat model of Parkinson disease and resulted in improved functional neuroprotection in the brain (Laganiere et al., 2010). Also, Lipofectamine-mediated transfection into human embryonic kidney cells indicated that ZFNs can be used to specifically target the ROSA26 safe site of the chromosome in order to effectively establish new cell lines with predictable expression level of the desired therapeutic gene (Perez-Pinera et al., 2012). In a different study, lipid-based nanoparticles were applied to deliver HR donor plasmids carrying the homologous sequence to hepatitis B virus (HBV) and specific ZFNs into cells that have been infected by HBV virus. This study confirmed the pharmaceutical application of ZFNs toward specific cleaving the invading virus genome in infected human cells as a new therapeutic approach (Cradick et al., 2010).

Like any other integrating system, application of ZFNs are limited to the very precise recognition of the target site and the homology sequence, precise design and construction of the appropriate ZFN, risk of non-specific and random binding of the ZFNs into the undesired sites with similar but not perfect homology and consequent integration of therapeutic transgene into unwanted locations, genotoxicity, and finally, the risk of leaving open ended double strand breaks and its consequent risks such as mutation, genome rearrangement, or cell death (Wirt and Porteus, 2012). Several investigations carried on to improve the specificity of ZFNs in clinically relevant cells. Some of these studies applied lipid-based nanoparticles to target cells and deliver donor DNA vectors to the target site. For example, a modular assembly technique was described to find the most effective ZFNs after delivery (Kim et al., 2009), or the “OPEN (Oligomerized Pool ENgineering)” technology was developed to construct over 30 highly active and effective ZFN pairs to target different chromosomal regions in human cells by lipofection (Maeder et al., 2008; Fu et al., 2009).

## TALEN

**Figure 5:**
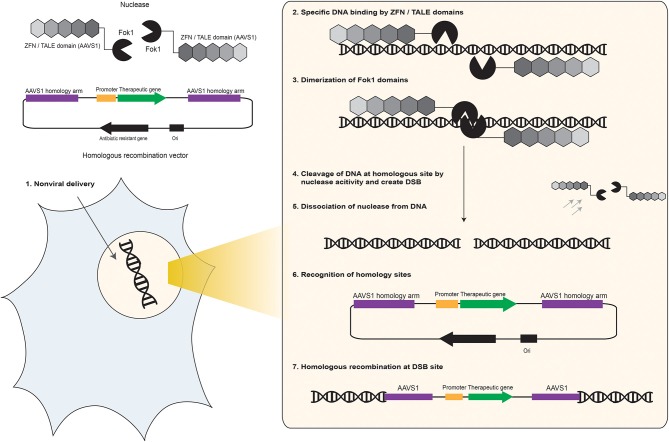
**Schematic representation of ZFN and TALEN-mediated homologous recombination into *AAVS1* safe site of human chromosome**. The FokI endonuclease enzyme derived from the prokaryote *Flavobacteriumokeanokoites* was found to function as two separate domains - one binds DNA in a sequence specific manner and one cleaves in a sequence independent manner that is highly specific because it needs dimerization for endonuclease activity on the target DNA. Therefore, by fusing a FokI monomer to two ZFPs or TALEs, which bind to adjacent sequences at a safe site on the human chromosome such as *AAVS1* target site, it is possible to generate sequence specific DNA nuclease complexes that facilitate selective targeting and homologous recombination within human genomes (Davis and Stokoe, 2010). (1) Two ZFPs or TALEs are designed to specifically target *AAVS1* site in anticipated clinically relevant target cell. These proteins are fused to a FokI cleavage domain to facilitate their nuclease activity. The ZFN or TALEN are co-delivered with the HR donor plasmid DNA vector into the cell by a non-viral gene delivery technique to reach the nuclei. (2) Inside the nucleus, ZFNs or TALENs recognize specific DNA sequences on the target cell's chromosome. (3) Binding of two ZFNs or TALENs complex to the target site allows FoKI to dimerize. (4) ZFNs or TALENs complex creates a targeted chromosome break at the *AAVS1* site of the host cell, which facilitates HR between the donor DNA vector and homologous sequences. (5) After generating the DNA double strand break, the ZFNs or TALENs complex disassociate from target DNA. (6) The AAVS1 homology arms subcloned into the HR donor DNA vectors recognize the homolog sequences located on the host genome. (7) HR-mediated insertion of the therapeutic gene expression cargo occurs at the *AAVS1* site of the clinically relevant host cell.

The TALENs are pre-designed restriction enzymes that are capable of specific cutting of a desired DNA sequence. However, similar to ZF or any other nuclease-based insertion method, customer/project-based design of these restriction enzymes is necessary and needs precise understanding and computer-based analysis of the target cell and insertion site because the off-target cleavage of potential similar sequences causes too much damage to the host genome and usually cause cell death. Very recently, applying the “Golden Gate TALEN and TAL Effector Kit” from Addgene, human embryonic kidney and human iPSC cells were genetically modified efficiently with TALEN system (Cermak, 2011). The “Golden-Gate assembly system” was developed using over 400 different plasmid DNA vector carrying TAL effector and Folk1 encoding genes in order to make a TALEN library. These plasmids complexed with Lipofectamine or polyethylenimine were delivered to several human cells to monitor their activity (Kim et al., 2013). However, similar to ZFN, TAL effector proteins require protein engineering to bind to a desired DNA sequence. Other improved system has been introduced by System Bioscience Company, which facilitates the precise insertion of the gene of interest into the *AAVS1* “safe site” of the human genome using the TALEN endonuclease technology. The *AAVS1* site, located on chromosome 19, is a natural integration hotspot of Adeno-associated virus (AAV) type 2 (Hüser and Heilbronn, 2003). It was shown that *AAVS1* site can be considered as a safe site for nuclease mediated HR and insertion of the therapeutic genes for stable and long-term expression (Sadelain et al., 2012). Recently, ZFNs and TALEN targeted to *AAVS1* were also used to insert therapeutic genes into this location (Figure 5), such as insertion of the transgene encoding gp91 in iPS cells derived from patients with X-linked chronic granulomatous disease and restore the gp91 function. These patients suffer from defective gp91 expression and are not able to produce reactive oxygen species (Zou et al., 2011). Insertion of exogenous gp91 into the *AAVS1* site is a novel pharmaceutical approach to cure this disease. In a different study, the activity of several exogenous and endogenous mammalian promoters on the expression levels of the transgenes were investigated applying plasmid-based TALEN strategy combined with nanoparticle-mediated transfection to insert promoters for more transient expression and check expression of the downstream genes over time (Smith, 2008). More studies on the effect of epigenetic regulations on transgene expression level, effect of promoter silencing, and transcription factors have been done using TALEN technology. For example, TALEN plasmid and transgene plasmids were delivered into human cells using Lipofectamine and polyethyleneimine nanoparticles to investigate selective activation of the endogenous *oct4* pluripotency gene *via* TALEN technology by activating reprogramming genes and inhibiting epigenetic factors to generate induced pluripotent stem cells (iPSCs) from somatic cells (Bultmann et al., 2012).

## CRISPR

CRISPR is the name of DNA sites in bacteria containing short sequences repeated multiple times within the genome of prokaryotes. These sequences produce adaptive immunity in bacterial cells against phage and plasmid infections and protect bacterial cells against invading viruses or foreign plasmids. The endonuclease encoding locus stores snippets of foreign sequence, which eventually would be transcribed into RNAs. These RNAs would be used as a guide based on sequences complementarity to introduce site-specific double-strand break in the target DNA or to cleave invading nucleic acids (Doudna and Charpentier, 2014). The endonuclease CRISPR-associated protein Cas9 cleaves DNA according to the sequence within an RNA duplex and creates site-specific double-strand break. CRISPRs are often associated with *cas* genes which code for endonucleases that perform various functions related to CRISPRs such as using a guide sequence within a RNA duplex and aligning it with a target DNA sequence. Post alignment, Cas9 generates a DSB in the target DNA, which provides the DNA open ends and initiate non-homologous recombination or homologous recombination if the similar sequence has been provided by a plasmid DNA vector or any other endogenousnucleic acid (Doudna and Charpentier, 2014). Recently, the CRISPR-Cas system has been adapted as a gene editing technique in mammalian cells by applying a DNA vector carrying *cas* genes, therapeutic transgene, and specifically designed CRISPRs to precisely cut host human cell genomic DNA at a desired location (Mali et al., 2013). However, for few years limited progress has been made to improve Cas specificity in variety of human cells due to target cell genotype heterogeneity. Unlike other nuclease methods, the CRISPR-Cas system requires the design and synthesis of a guide RNA that simultaneously targets multiple genomic sites. In last few years, the CRISPR-Cas technology in combination with better delivery systems such as physical methods and more advanced nanocarriers dramatically improved its specificity and efficacy. The number of publications that applied this technology to edit the genome of human somatic cells, stem cells, and iPSC cells has been increased radically and several companies are offering commercially available kits and services to assist scientists with multidisciplinary backgrounds who are interested in applying this technique. The following are just few examples to demonstrate the increasing popularity of this technology among clinical and scientific researchers. CRISPR-Cas system has been used to (A) provide in-depth understanding of carcinogenesis processes through generating tumor-associated genomic rearrangements by causing DSB and non-homologous recombination of the DNA open ends (Choi and Meyerson, 2014; Torres et al., 2014); (B) facilitate systematic analysis of gene functions in human cells by activation (Cheng et al., 2013) or inactivation of essential genes for positive or negative selection (Wang et al., 2014) or genes that play key roles in cell viability and cancer progression, reprogramming somatic cells to generate iPSC cells (Shalem et al., 2014), and large-scaled genetic screening for drug targets (Doudna and Charpentier, 2014); (C) facilitate gene therapy of genetic diseases by correcting genetic mutations involved in inherited disorders by HR (Wu et al., 2013); (D) produce genetically engineered animal models for different human diseases and drug discovery (Ma et al., 2014; Wang et al., 2014); (E) develop new antiviral treatments by making DSB in the provirus genomic DNA integrated into the infected host cell genome and deactivating the viral genes necessary for pathogenesis or reproduction, reassembly and release of new viruses (Ebina et al., 2013; Hu et al., 2014); and (F) facilitate precise *ex vivo* remodeling of stem/progenitor cells (Doudna and Charpentier, 2014).

## Conclusions

More advanced and safer techniques for genome editing have many potential applications in further understanding of neurodegenerative diseases, including (A) generating new cell lines to study basics of disease progression, cell growth, and differentiation; (B) screening, identification, and developments of novel therapeutics and their targets; (C) generating disease animal models; and (D) developing new candidates for cell replacement or cell rescue therapies (Yin et al., 2014a).

Neurotrophic factor therapeutic genome engineering is a highly coveted clinical aspiration of glaucoma therapy, but only recent technological advances in improved delivery techniques, the new generation of bacterial sequence-depleted DNA vectors carrying only the desired therapeutic transgene expression cargo, and the availability of modern genome editing techniques could provide the necessary tools for the modification of complex genomes and gene therapy in a targeted fashion. We previously highlighted the rationale for NTFs and their transgene delivery into cell candidates and discussed the significant effects of several NTFs on the support and protection of retinal cells and other cell candidates that might be potential therapeutic options for glaucoma neuroprotective gene therapy (Nafissi and Foldvari, 2015). Here, we highlighted the rationale of genetic modifications for glaucoma therapies either as the gene therapy targets or cell replacement therapy to provide long-term neurotrophin therapeutics on-site using new generations of safer and more efficient DNA vectors and modern genetic modification techniques.

In general, compared to cell replacement therapies to replace and integrate functionally active stem/progenitor cells into the retina, it was shown that cell-based neuroprotective therapies by prolonged secretion of NTFs is a more straightforward strategy to nourish and support dying RGCs in progressive glaucoma. In addition to supplying NTFs, cell-based neuroprotective therapies may also be able to promote RGCs survival directly through modifying gene expression in surrounding cells to enhance NTFs expression on-site (Madhavan et al., 2008). Genetically modified retinal progenitor cells, that express and secrete the NTFs, have been shown to provide neuroprotective support to degenerated or injured RGCs similar to the supportive role of NTFs expressing NSC or mesenchymal stem cells (MSCs) in regeneration of neurons (Akerud et al., 2001; Nomura et al., 2005; Zhu et al., 2005).

Thus, genetically modified NTFs secreting cell-based therapies provides new avenues to fight with the irreversible loss of RGCs associated with glaucoma. Currently we have efficient tools for permanent, reversible, or conditional genetic manipulations of almost any type of cells. The success of clinical trials in other disorders gives rise to similar approaches in the context of neurodegenerative diseases including glaucoma. Ultimately, a better understanding of cell development and stem cell biology at the molecular level, along with improvements in the techniques for non-viral gene delivery, advances in nanotechnology and nanomedicine for synthesis of more cell-friendly structures, and in-depth knowledge of gene modification systems at the molecular level would encourage scientists to develop novel treatments for degenerative disorders. Non-viral gene delivery techniques such as electroporation (Helledie et al., 2008) nucleofection (Dwivedi et al., 2012; Madeira et al., 2013), as well as nanotechnology tools such as application of polysaccharide nanoparticles (Deng et al., 2013), cationic liposome nanoparticles (Madeira et al., 2010), calcium phosphate nanoparticles (Cao et al., 2011), and nanoneedles (Nakamura et al., 2002), in parallel with efficient and safe therapeutic transgene insertion techniques such as ΦC31 integrase, transposons, and ZFN, TALEN, or the CRISPR/Cas9 nuclease systems (Gaj et al., 2013), are new tools for developing clinically approved cell-based therapeutics for neurodegenerative disorders like glaucoma. These systems are more reliable since they avoid the high risks associated with using viral vectors such as insertional mutagenesis and undesired immune rejection, provide life-long therapy by more policed insertion of the therapeutic gene into the desired site, target a broader range of disorders due to their capability to accommodate genes of different sizes, and finally, provides higher activity owing to their ability to target hard- to-transfect human cells. Before *ex vivo* modified cells can achieve therapeutic efficacy for clinical glaucoma treatment in human, many limitations remain to be overcome. A reliable and expandable source of retinal cells must be isolated, expanded, characterized and established. These cells must demonstrate appropriate engraftment within the retina. Even with the aforementioned challenges, the *in vitro, ex vivo*, and pre-clinical support for genetically modified cell-mediated RGC repair is undeniable, and warrants the kind of robust investigation that is currently under way. These methods, if successful in human trials, could offer several advantages over traditional pharmacological approaches. While much work remains to address the safety issues for all of the above mentioned approaches, evidently, this is a multi-disciplinary field which undeniably needs collaboration from different scientific domains to bring the bench results to the clinic.

### Conflict of interest statement

The authors declare that the research was conducted in the absence of any commercial or financial relationships that could be construed as a potential conflict of interest.

## References

[B1] Akerud P. Canals J. M. Snyder E. Y. Arenas E. (2001). Neuroprotection through delivery of glial cell line-derived neurotrophic factor by neural stem cells in a mouse model of Parkinson's disease. *J*. Neurosci. 21, 8108–8118. 11588183 10.1523/JNEUROSCI.21-20-08108.2001 PMC6763865

[B2] Aronovich E. L. McIvor R. S. Hackett P. B. (2011). The Sleeping beauty transposon system: a non-viral vector for gene therapy. Hum. Mol. Genet. 20, R14–R20. 10.1093/hmg/ddr140 21459777 PMC3095056

[B3] Benowitz L. I. Yin Y. (2010). Optic nerve regeneration. Arch. Ophthalmol. 128, 1059–1064. 10.1001/archophthalmol.2010.152 20697009 PMC3072887

[B4] Benowitz L. Yin Y. (2008). Rewiring the injured CNS: lessons from the optic nerve. Exp. Neurol. 209, 389–398. 10.1016/j.expneurol.2007.05.025 17610877 PMC2323976

[B5] Bertoni C. Jarrahian S. Wheeler T. M. Li Y. Olivares E. C. Calos M. P. (2006). Enhancement of plasmid-mediated gene therapy for muscular dystrophy by directed plasmid integration. Proc. Natl. Acad. Sci. U.S.A. 103, 419–424. 10.1073/pnas.0504505102 16387861 PMC1326153

[B6] Bessero A. C. Clarke P. G. (2010). Neuroprotection for optic nerve disorders. Curr. Opin. Neurol. 23, 10–15. 10.1097/WCO.0b013e3283344461 19915465

[B7] Bigger B. W. (2001). An araC-controlled bacterial cre expression system to produce DNA minicircle vectors for nuclear and mitochondrial gene therapy. J. Biol. Chem. 276, 23018–23027. 10.1074/jbc.M010873200 11304530

[B8] Blurton-Jones M. Spencer B. Michael S. Castello N. A. Agazaryan A. A. Davis J.. (2014). Neural stem cells genetically-modified to express neprilysin reduce pathology in Alzheimer transgenic models. Stem Cell Res. Ther. 5, 1–14. 10.1186/scrt440 25022790 PMC4055090

[B9] Bultmann S. Morbitzer R. Schmidt C. S. Thanisch K. Spada F. Elsaesser J.. (2012). Targeted transcriptional activation of silent oct4 pluripotency gene by combining designer TALEs and inhibition of epigenetic modifiers. Nucleic Acids Res. 40, 5368–5377. 10.1093/nar/gks199 22387464 PMC3384321

[B10] Calos M. P. (2006). The phiC31 integrase system for gene therapy. Curr. Gene Ther. 6, 633–645. 10.2174/156652306779010642 17168696

[B11] Cao X. Deng W. Yuan Y. Weiyan S. Yan Y. Yawei W.. (2011). Encapsulation of plasmid DNA in calcium phosphate nanoparticles: stem cell uptake and gene transfer efficiency. Int. J. Nanomedicine 6, 3335. 10.2147/IJN.S27370 22229000 PMC3252680

[B12] Cavagnaro J. A. (2013). Preclinical Safety Evaluation of Biopharmaceuticals: A Science-Based Approach to Facilitating Clinical Trials. John Wiley & Sons.

[B13] Cermak T. (2011). Efficient design and assembly of custom TALEN and other TAL effector-based constructs for DNA targeting. Nucleic Acids Res. 39:e82. 10.1093/nar/gkr218 21493687 PMC3130291

[B14] Chakraborty S. Ji H. Chen J. Gersbach C. A. Leong K. W. (2014). Vector modifications to eliminate transposase expression following piggyBac-mediated transgenesis. Sci. Rep. 4:7403. 10.1038/srep07403 25492703 PMC4261183

[B15] Chalberg T. W. Portlock J. L. Olivares E. C. Thyagarajan B. Kirby P. J. Hillman R. T. (2006). Integration specificity of phage phiC31 integrase in the human genome. J. Mol. Biol. 357, 28–48. 10.1016/j.jmb.2005.11.098 16414067

[B16] Chavez C. L. Keravala A. Chu J. N. Farruggio A. P. Cuéllar V. E. Voorberg J.. (2011). Long-term expression of human coagulation factor VIII in a tolerant mouse model using the φC31 integrase system. Hum. Gene Ther. 23, 390–398. 10.1089/hum.2011.110 22077817 PMC3327602

[B17] Chen J. Rattner A. Nathans J. (2005). The rod photoreceptor-specific nuclear receptor Nr2e3 represses transcription of multiple cone-specific genes. J. Neurosci. 25, 118–129. 10.1523/JNEUROSCI.3571-04.2005 15634773 PMC6725199

[B18] Chen Z. Y. He C-Y. Ehrhardt A. Kay M. A. (2003). Minicircle DNA vectors devoid of bacterial DNA result in persistent and high-level transgene expression *in vivo*. Mol. Ther. 8, 495–500. 10.1016/S1525-0016(03)00168-0 12946323

[B19] Chen Z. Y. Riu E. He C. Y. Xu H. Kay M. A. (2008). Silencing of episomal transgene expression in liver by plasmid bacterial backbone DNA is independent of CpG methylation. Mol. Ther. 16, 548–556. 10.1038/sj.mt.6300399 18253155

[B20] Cheng A. W. Wang H. Yang H. Shi L. Katz Y. Theunissen T.. (2013). Multiplexed activation of endogenous genes by CRISPR-on, an RNA-guided transcriptional activator system. Cell Res. 23, 1163–1171. 10.1038/cr.2013.122 23979020 PMC3790238

[B21] Choi P. S. Meyerson M. (2014). Targeted genomic rearrangements using CRISPR/Cas technology. Nat. Commun. 5:3728. 10.1038/ncomms4728 24759083 PMC4170920

[B22] Cradick T. J. Keck K. Bradshaw S. Jamieson A. C. McCaffrey A. P. (2010). Zinc-finger nucleases as a novel therapeutic strategy for targeting hepatitis B Virus DNAs. Mol. Ther. 18, 947–954. 10.1038/mt.2010.20 20160705 PMC2890117

[B23] Crigler L. Robey R. C. Asawachaicharn A. Gaupp D. Phinney D. G. (2006). Human mesenchymal stem cell subpopulations express a variety of neuro-regulatory molecules and promote neuronal cell survival and neuritogenesis. Exp. Neurol. 198, 54–64. 10.1016/j.expneurol.2005.10.029 16336965

[B24] Danesh-Meyer H. V. (2011). Neuroprotection in glaucoma: recent and future directions. Curr. Opin. Ophthalmol. 22, 78–86. 10.1097/ICU.0b013e32834372ec 21252670

[B25] Darquet A. M. Cameron B. Wils P. Scherman D. Crouzet J. (1997). A new DNA vehicle for nonviral gene delivery: supercoiled minicircle. Gene Ther. 4, 1341–1349. 9472558 10.1038/sj.gt.3300540

[B26] Darquet A. M. Rangara R. Kreiss P. Schwartz B. Naimi S. Delaère P. (1999). Minicircle: an improved DNA molecule for *in vitro* and *in vivo* gene transfer. Gene Ther. 6, 209–218. 10435105 10.1038/sj.gt.3300816

[B27] Davis D. Stokoe D. (2010). Zinc Finger Nucleases as tools to understand and treat human diseases. BMC Med. 8:42. 10.1186/1741-7015-8-42 20594338 PMC2904710

[B28] Davis R. P. Nemes C. Varga E. Freund C. Kosmidis G. Gkatzis K.. (2013). Generation of induced pluripotent stem cells from human foetal fibroblasts using the Sleeping Beauty transposon gene delivery system. Differentiation 86, 30–37. 10.1016/j.diff.2013.06.002 23933400

[B29] Deng W. Fu M. Cao Y. Cao X. Wang M. Yang Y.. (2013). Angelica sinensis polysaccharide nanoparticles as novel non-viral carriers for gene delivery to mesenchymal stem cells. Nanomedicine 9, 1181–1191. 10.1016/j.nano.2013.05.008 23727125

[B30] Di Matteo M. Belay E. Chuah M. K. Vandendriessche T. (2012). Recent developments in transposon-mediated gene therapy. Expert Opin. Biol. Ther. 12, 841–858. 10.1517/14712598.2012.684875 22679910

[B31] Di Polo A. Aigner L. J. Dunn R. J. Bray G. M. Aguayo A. J. (1998). Prolonged delivery of brain-derived neurotrophic factor by adenovirus-infected Muller cells temporarily rescues injured retinal ganglion cells. Proc. Natl. Acad. Sci. U.S.A. 95, 3978–3983. 9520478 10.1073/pnas.95.7.3978 PMC19948

[B32] Doudna J. A. Charpentier E. (2014). The new frontier of genome engineering with CRISPR-Cas9. Science 346, 1077. 10.1126/science.1258096 25430774

[B33] Dwivedi P. P. Anderson P. J. Powell B. C. (2012). Development of an efficient, non-viral transfection method for studying gene function and bone growth in human primary cranial suture mesenchymal cells reveals that the cells respond to BMP2 and BMP3. BMC Biotechnol. 12:45. 10.1186/1472-6750-12-45 22857382 PMC3431223

[B34] Ebina H. Misawa N. Kanemura Y. Koyanagi Y. (2013). Harnessing the CRISPR/Cas9 system to disrupt latent HIV-1 provirus. Sci. Rep. 3:2510. 10.1038/srep02510 23974631 PMC3752613

[B35] Fang J. H. Wang X. H. Xu Z. R. Jiang F. G. (2010). Neuroprotective effects of bis(7)-tacrine against glutamate-induced retinal ganglion cells damage. BMC Neurosci. 11:31. 10.1186/1471-2202-11-31 20199668 PMC2838896

[B36] Foster A. Resnikoff S. (2005). The impact of Vision 2020 on global blindness. Eye 19, 1133–1135. 10.1038/sj.eye.6701973 16304595

[B37] Fu F. Sander J. D. Maeder M. Thibodeau-Beganny S. Joung J. K. Dobbs D. (2009). Zinc Finger Database (ZiFDB): a repository for information on C2H2 zinc fingers and engineered zinc-finger arrays. Nucleic Acids Res. 37, D279–D283. 10.1093/nar/gkn606 18812396 PMC2686427

[B38] Gaj T. Gersbach C. A. Barbas C. F. (2013). ZFN, TALEN, and CRISPR/Cas-based methods for genome engineering. Trends Biotechnol. 31, 397–405. 10.1016/j.tibtech.2013.04.004 23664777 PMC3694601

[B39] Gorelik M. Orukari I. Wang J. Galpoththawela S. Kim H. Levy M.. (2012). Use of MR cell tracking to evaluate targeting of glial precursor cells to inflammatory tissue by exploiting the very late antigen-4 docking receptor. Radiology 265, 175–185. 10.1148/radiol.12112212 22923719 PMC3447172

[B40] Groth A. C. Calos M. P. (2004). Phage integrases: biology and applications. J. Mol. Biol. 335, 667–678. 10.1016/j.jmb.2003.09.082 14687564

[B41] Groth A. C. Olivares E. C. Thyagarajan B. Calos M. P. (2000). A phage integrase directs efficient site-specific integration in human cells. Proc. Natl. Acad. Sci. U.S.A. 97, 5995–6000. 10.1073/pnas.090527097 10801973 PMC18547

[B42] Hackett P. B. Jr. Aronovich E. L. Hunter D. Urness M. Bell J. B. Kass S. J.. (2011). Efficacy and safety of sleeping beauty transposon-mediated gene transfer in preclinical animal studies. Curr. Gene Ther. 11, 341–349. 10.2174/156652311797415827 21888621 PMC3728161

[B43] Held P. K. Olivares E. C. Aguilar C. P. Finegold M. Calos M. P. Grompe M. (2005). In vivo correction of murine hereditary tyrosinemia type I by phiC31 integrase-mediated gene delivery. Mol. Ther. 11, 399–408. 10.1016/j.ymthe.2004.11.001 15727936

[B44] Helledie T. Nurcombe V. Cool S. M. (2008). A simple and reliable electroporation method for human bone marrow mesenchymal stem cells. Stem Cells Dev. 17, 837–848. 10.1089/scd.2007.0209 18752428

[B45] Hüser D. Heilbronn R. (2003). Adeno-associated virus integrates site-specifically into human chromosome 19 in either orientation and with equal kinetics and frequency. J. Gen. Virol. 84, 133–137. 10.1099/vir.0.18726-0 12533709

[B46] Hu W. Kaminski R. Yang F. Zhang Y. Cosentino L. Li F.. (2014). RNA-directed gene editing specifically eradicates latent and prevents new HIV-1 infection. Proc. Natl. Acad. Sci. U.S.A. 111, 11461–11466. 10.1073/pnas.1405186111 25049410 PMC4128125

[B47] Iwabe S. Moreno-Mendoza N. A. Trigo-Tavera F. Crowder C. Garcia-Sánchez G. A. (2007). Retrograde axonal transport obstruction of brain-derived neurotrophic factor (BDNF) and its TrkB receptor in the retina and optic nerve of American Cocker Spaniel dogs with spontaneous glaucoma. Vet. Ophthalmol. 10, 12–19. 10.1111/j.1463-5224.2007.00504.x 17973830

[B48] Jakobsen J. E. Johansen M. G. Schmidt M. Dagnæs-Hansen F. Dam K. Gunnarsson A.. (2013). Generation of minipigs with targeted transgene insertion by recombinase-mediated cassette exchange (RMCE) and somatic cell nuclear transfer (SCNT). Transgenic Res. 22, 709–723. 10.1007/s11248-012-9671-6 23111619 PMC3712138

[B49] Jamieson A. C. Miller J. C. Pabo C. O. (2003). Drug discovery with engineered zinc-finger proteins. Nat. Rev. Drug Discov. 2, 361–368. 10.1038/nrd1087 12750739

[B50] Jandial R. Singec I. Ames C. P. Snyder E. Y. (2008). Genetic modification of neural stem cells. Mol. Ther. 16, 450–457. 10.1038/sj.mt.6300402 18253153

[B51] Janowski M. Date I. (2011). Systemic neurotransplantation - A problem-oriented systematic review. Rev. Neurosci. 20, 39–60. 10.1515/REVNEURO.2009.20.1.39 19526733

[B52] Ji J. Z. Elyaman W. Yip H. K. Lee V. W. Yick L. W. Hugon J.. (2004). CNTF promotes survival of retinal ganglion cells after induction of ocular hypertension in rats: the possible involvement of STAT3 pathway. Eur. J. Neurosci. 19, 265–272. 10.1111/j.0953-816X.2003.03107.x 14725620

[B53] Jia F. Wilson K. D. Sun N. Gupta D. M. Huang M. Li Z.. (2010). A nonviral minicircle vector for deriving human iPS cells. Nat. Methods 7, 197–199. 10.1038/nmeth.1426 20139967 PMC2892897

[B54] Jiang C. Moore M. J. Zhang X. Klassen H. Langer R. Young M. (2007). Intravitreal injections of GDNF-loaded biodegradable microspheres are neuroprotective in a rat model of glaucoma. Mol. Vis. 13, 1783–1792. 17960131

[B55] Johnen S. Izsvák Z. Stöcker M. Harmening N. Salz A. K. Walter P.. (2012). Sleeping beauty transposon-mediated transfection of retinal and iris pigment epithelial cells. Invest. Ophthalmol. Vis. Sci. 53, 4787–4796. 10.1167/iovs.12-9951 22729435

[B56] Johnson E. C. Morrison J. C. (2009). Friend or foe? Resolving the impact of glial responses in glaucoma. J. Glaucoma 18, 341–353. 10.1097/IJG.0b013e31818c6ef6 19525723 PMC2697444

[B57] Johnson T. V. Bull N. D. Martin K. R. (2011). Neurotrophic factor delivery as a protective treatment for glaucoma. Exp. Eye Res. 93, 196–203. 10.1016/j.exer.2010.05.016 20685205

[B58] Kachi S. Esumi N. Zack D. J. Campochiaro P. A. (2006). Sustained expression after nonviral ocular gene transfer using mammalian promoters. Gene Ther. 13, 798–804. 10.1038/sj.gt.3302700 16467860

[B59] Kakarougkas A. Jeggo P. A. (2014). DNA DSB repair pathway choice: an orchestrated handover mechanism. Br. J. Radiol. 87:20130685. 10.1259/bjr.20130685 24363387 PMC4064598

[B60] Kay M. A. (2011). State-of-the-art gene-based therapies: the road ahead. Nat. Rev. Genet. 12, 316–328. 10.1038/nrg2971 21468099

[B61] Kim H. J. Lee H. J. Kim H. Cho S. W. Kim J. S. (2009). Targeted genome editing in human cells with zinc finger nucleases constructed via modular assembly. Genome Res. 19, 1279–1288. 10.1101/gr.089417.108 19470664 PMC2704428

[B62] Kim H. Kim J. S. (2014). A guide to genome engineering with programmable nucleases. Nat. Rev. Genet. 15, 321–334. 10.1038/nrg3686 24690881

[B63] Kim H. Um E. Cho S. R. Jung C. Kim H. Kim J. (2011). Surrogate reporters for enrichment of cells with nuclease-induced mutations. Nat. Methods 8, 941–943. 10.1038/nrg3686 21983922

[B64] Kim Y. Kweon J. Kim A. Chon J. K. Yoo J. Y. Kim H. J. (2013). A library of TAL effector nucleases spanning the human genome. Nat. Biotechnol. 31, 251–258. 10.1038/nbt.2517 23417094

[B65] Klinman D. M. Yi A. K. Beaucage S. L. Conover J. Krieg A. M. (1996). CpG motifs present in bacteria DNA rapidly induce lymphocytes to secrete interleukin 6, interleukin 12, and interferon gamma. Proc. Natl. Acad. Sci. U.S.A. 93, 2879–2883. 8610135 10.1073/pnas.93.7.2879 PMC39727

[B66] Kobelt D. Schleef M. Schmeer M. Aumann J. Schlag P. Walther W. (2013). Performance of high quality minicircle DNA for *in vitro* and *in vivo* gene transfer. Mol. Biotechnol. 53, 80–89. 10.1007/s12033-012-9535-6 22467123

[B67] Laganiere J. Kells A. P. Lai J. T. Guschin D. Paschon D. E. Meng X. Fong L. K.. (2010). An engineered zinc finger protein activator of the endogenous glial cell line-derived neurotrophic factor gene provides functional neuroprotection in a rat model of Parkinson's disease. J. Neurosci. 30, 16469–16474. 10.1523/JNEUROSCI.2440-10.2010 21147986 PMC6634881

[B68] Lan F. Liu J. Narsinh K. H. Hu S. Han L. Lee A. S.. (2012). Safe genetic modification of cardiac stem cells using a site-specific integration technique. Circulation 126(11 Suppl. 1), S20–S28. 10.1161/CIRCULATIONAHA.111.084913 22965984 PMC3481839

[B69] Li M. A. Turner D. J. Ning Z. Yusa K. Liang Q. Eckert S. (2011). Mobilization of giant piggyBac transposons in the mouse genome. Nucleic Acids Res. 39:e148. 10.1093/nar/gkr764 21948799 PMC3239208

[B70] Li R. Zhuang Y. Han M. Xu T. Wu X. (2013). piggyBac as a high-capacity transgenesis and gene-therapy vector in human cells and mice. Dis. Model. Mech. 6, 828–833. 10.1242/dmm.010827 23519027 PMC3634665

[B71] Lim S. T. Airavaara M. Harvey B. K. (2010). Viral vectors for neurotrophic factor delivery: a gene therapy approach for neurodegenerative diseases of the CNS. Pharmacol. Res. 61, 14–26. 10.1016/j.phrs.2009.10.002 19840853 PMC2880921

[B72] Liu J Skjørringe, T. Gjetting T. Jensen T. G. (2009). PhiC31 integrase induces a DNA damage response and chromosomal rearrangements in human adult fibroblasts. BMC Biotechnol. 9:31. 10.1186/1472-6750-9-31 19341467 PMC2682486

[B73] Ma K. Wang D.-D. Lin Y. Wang J. Petrenko V. Mao C. (2013). Synergetic targeted delivery of sleeping-beauty transposon system to mesenchymal stem cells using LPD nanoparticles modified with a phage-displayed targeting peptide. Adv. Funct. Mater. 23, 1172–1181. 10.1002/adfm.201102963 23885226 PMC3718568

[B74] Ma Y. Shen B. Zhang X. Lu Y. Chen W. Ma J.. (2014). Heritable multiplex genetic engineering in rats using CRISPR/Cas9. PLoS ONE 9:e89413. 10.1371/journal.pone.0089413 24598943 PMC3943732

[B75] Madeira C. Mendes R. D. Ribeiro S. C. Boura J. S. Aires-Barros M. R. da Silva C. L.. (2010). Nonviral gene delivery to mesenchymal stem cells using cationic liposomes for gene and cell therapy. Biomed Res. Int. 2010:735349. 10.1155/2010/735349 20625411 PMC2896879

[B76] Madeira C. Rodrigues C. A. Reis M. S. Ferreira F. F. Correia R. E. Diogo M. M.. (2013). Nonviral gene delivery to neural stem cells with minicircles by microporation. Biomacromolecules 14, 1379–1387. 10.1021/bm400015b 23514247

[B77] Madhavan L. Ourednik V. Ourednik J. (2008). Neural stem/progenitor cells initiate the formation of cellular networks that provide neuroprotection by growth factor-modulated antioxidant expression. Stem Cells 26, 254–265. 10.1634/stemcells.2007-0221 17962704

[B78] Maeder M. L. Thibodeau-Beganny S. Osiak A. Wright D. A. Anthony R. M. Eichtinger M.. (2008). Rapid “open-source” engineering of customized zinc-finger nucleases for highly efficient gene modification. Mol. Cell 31, 294–301. 10.1016/j.molcel.2008.06.016 18657511 PMC2535758

[B79] Mali P. Yang L. Esvelt K. M. Aach J. Guell M. DiCarlo J. E.. (2013). RNA-guided human genome engineering via Cas9. Science 339, 823–826. 10.1126/science.1232033 23287722 PMC3712628

[B80] Margalit E. Sadda S. R. (2003). Retinal and optic nerve diseases. Artif. Organs 27, 963–974. 10.1046/j.1525-1594.2003.07304 14616515

[B81] Mátés L. Chuah M. K. L. Belay E. Jerchow B. Manoj N. Acosta-Sanchez A.. (2009). Molecular evolution of a novel hyperactive Sleeping Beauty transposase enables robust stable gene transfer in vertebrates. Nat. Genet. 41, 753–761. 10.1038/ng.343 19412179

[B82] Mayrhofer P. Schleef M. Jechlinger W. (2009). Use of minicircle plasmids for gene therapy. Methods Mol. Biol. 542, 87–104. 10.1007/978-1-59745-561-9_4 19565897

[B83] Miller A. M. Dean D. A. (2009). Tissue-specific and transcription factor-mediated nuclear entry of DNA. Adv. Drug Deliv. Rev. 61, 603–613. 10.1016/j.addr.2009.02.008 19393704

[B84] Mitsui M. Nishikawa M. Zang L. Ando M. Hattori K. Takahashi Y.. (2009). Effect of the content of unmethylated CpG dinucleotides in plasmid DNA on the sustainability of transgene expression. J. Gene Med. 11, 435–443. 10.1002/jgm.1317 19291673

[B85] Nafissi N. Alqawlaq S. Lee E. A. Foldvari M. Spagnuolo P. A. Slavcev R. A. (2014). DNA ministrings: highly safe and effective gene delivery vectors. Mol. Ther. Nucleic Acids 3:e165. 10.1038/mtna.2014.16 24892724 PMC4078758

[B86] Nafissi N. Foldvari M. (2015). Neuroprotective therapies in glaucoma: I. Neurotrophic factor delivery. Wiley Interdiscip. Rev. Nanomed. Nanobiotechnol. [Epub ahead of print]. 10.1002/wnan.1361 26306832

[B87] Nafissi N. Slavcev R. (2012). Construction and characterization of an *in-vivo* linear covalently closed DNA vector production system. Microb. Cell Fact. 11:154. 10.1186/1475-2859-11-154 23216697 PMC3540006

[B88] Nafissi N. Slavcev R. (2014). Bacteriophage recombination systems and biotechnical applications. Appl. Microbiol. Biotechnol. 98, 2841–2851. 10.1007/s00253-014-5512-2 24442504

[B89] Nakamura T. Sato K. Hamada H. (2002). Effective gene transfer to human melanomas via integrin-targeted adenoviral vectors. *Hum*. Gene Ther. 13, 613–626. 10.1089/10430340252837215 11916485

[B90] Nakazawa Y. Huye L. E. Dotti G. Foster A. E. Vera J. F. Manuri P. R.. (2009). Optimization of the PiggyBac transposon system for the sustained genetic modification of human T-Lymphocytes. J. Immunother. 32, 826–836. 10.1097/CJI.0b013e3181ad762b 19752751 PMC2796278

[B91] Nienhuis A. W. (2008). Development of gene therapy for blood disorders. Blood 111, 4431–4444. 10.1182/blood-2007-11-078121 18441245

[B92] Nomura T. Honmou O. Harada K. Houkin K. Hamada H. Kocsis J. D. (2005). I.V. infusion of brain-derived neurotrophic factor gene-modified human mesenchymal stem cells protects against injury in a cerebral ischemia model in adult rat. Neuroscience 136, 161–169. 10.1016/j.neuroscience.2005.06.062 16229956 PMC2605391

[B93] Oliveira A. V. V. Silva G. A. Chung D. C. (2015). Enhancement of chitosan-mediated gene delivery through combination with phiC31 integrase. Acta Biomater. 17, 89–97. 10.1016/j.actbio.2015.01.013 25600399

[B94] Palavesam A. Esnault C. O'Brochta D. A. (2013). Post-integration silencing of piggyBac transposable elements in *Aedes aegypti*. PLoS ONE 8:e68454. 10.1371/journal.pone.0068454 23861905 PMC3701635

[B95] Pan Y. Xiao L. Li A. S. Zhang X. Sirois P. Zhang J.. (2013). Biological and biomedical applications of engineered nucleases. Mol. Biotechnol. 55, 54–62. 10.1007/s12033-012-9613-9 23089945

[B96] Perez-Pinera P. Ousterout D. G. Brown M. T. Gersbach C. A. (2012). Gene targeting to the ROSA26 locus directed by engineered zinc finger nucleases. Nucleic Acids Res. 40, 3741–3752. 10.1093/nar/gkr1214 22169954 PMC3333879

[B97] Porteus M. (2008). Design and testing of zinc finger nucleases for use in mammalian cells, in Chromosomal Mutagenesis, Vol. 435, eds Davis G. Kayser K. (Humana Press), 47–61. 10.1007/978-1-59745-232-8_4 18370067

[B98] Porteus M. H. (2006). Mammalian gene targeting with designed zinc finger nucleases. Mol. Ther. 13, 438–446. 10.1016/j.ymthe.2005.08.003 16169774

[B99] Porteus M. H. Baltimore D. (2003). Chimeric nucleases stimulate gene targeting in human cells. Science 300:763. 10.1126/science.1078395 12730593

[B100] Rodríguez E. G. (2004). Nonviral DNA vectors for immunization and therapy: design and methods for their obtention. J. Mol. Med. 82, 500–509. 10.1007/s00109-004-0548-x 15175860

[B101] Sadelain M. Papapetrou E. P. Bushman F. D. (2012). Safe harbours for the integration of new DNA in the human genome. Nat. Rev. Cancer 12, 51–58. 10.1038/nrc3179 22129804

[B102] Sadowski P. (1986). Site-specific recombinases: changing partners and doing the twist. J. Bacteriol. 165, 341–347. 3003022 10.1128/jb.165.2.341-347.1986 PMC214421

[B103] Saha S. Woodard L. E. Charron E. M. Welch R. C. Rooney C. M. Wilson M. H. (2015). Evaluating the potential for undesired genomic effects of the piggyBac transposon system in human cells. Nucleic Acids Res. 43, 1770–1782. 10.1093/nar/gkv017 25605795 PMC4330379

[B104] Schakowski F. Gorschlüeter M. Buttgereit P. Maerten A. Lilienfeld-Toal M. V. Junghans C.. (2007). Minimal size MIDGE vectors improve transgene expression *in vivo*. In Vivo 21, 17–23. 17354609

[B105] Schakowski F. Gorschluter M. Junghans C. Schroff M. Buttgereit P. Ziske C.. (2001). A novel minimal-size vector (MIDGE) improves transgene expression in colon carcinoma cells and avoids transfection of undesired DNA. Mol. Ther. 3, 793–800. 10.1006/mthe.2001.0322 11356084

[B106] Shalem O. Sanjana N. E. Hartenian E. Shi X. Scott D. A. Mikkelsen T. S.. (2014). Genome-scale CRISPR-Cas9 knockout screening in human cells. Science 343, 84–87. 10.1126/science.1247005 24336571 PMC4089965

[B107] Sharma N. Cai Y. Bak R. O. Jakobsen M. R. Schrøder L. D. Mikkelsen J. G. (2013). Efficient sleeping beauty DNA transposition from DNA minicircles. Mol. Ther. Nucleic Acids 2:e74. 10.1038/mtna.2013.1 23443502 PMC3586802

[B108] Sivalingam J. Krishnan S. Ng W. H. Lee S. S. Phan T. T. Kon O. L. (2010). Biosafety assessment of site-directed transgene integration in human umbilical cord-lining cells. Mol. Ther. 18, 1346–1356. 10.1038/mt.2010.61 20424600 PMC2911251

[B109] Slavcev R. A. Nafissi N. (2014). DNA Vector Production System. United State of America, Google Patents. Application 14/087,715.

[B110] Slavcev R. Sum C. Nafissi N. (2014). Optimized production of a safe and efficient gene therapeutic vaccine versus HIV via a linear covalently closed DNA minivector. BMC Infect. Dis. 14:P74 10.1186/1471-2334-14-S2-P74

[B111] Smith J. R. (2008). Robust, persistent transgene expression in human embryonic stem cells is achieved with AAVS1-targeted integration. Stem Cells 26, 496–504. 10.1634/stemcells.2007-0039 18024421

[B112] Solodushko V. Bitko V. Fouty B. (2014). Minimal piggyBac vectors for chromatin integration. Gene Ther. 21, 1–9. 10.1038/gt.2013.52 24131979

[B113] Thumann G. (2012). Prospectives for Gene Therapy of Retinal Degenerations. Curr. Genomics 13, 350–362. 10.2174/138920212801619214 23372421 PMC3401892

[B114] Thyagarajan B. Liu Y. Shin S. Lakshmipathy U. Scheyhing K. Xue H. (2008). Creation of engineered human embryonic stem cell lines using phiC31 integrase. Stem Cells 26, 119–126. 10.1634/stemcells.2007-0283 17962703

[B115] Torres R. Martin M. C. Garcia A. Cigudosa J. C. Ramirez J. C. Rodriguez-Perales S. (2014). Engineering human tumour-associated chromosomal translocations with the RNA-guided CRISPR–Cas9 system. Nat. Commun. 5:3964. 10.1038/ncomms4964 24888982

[B116] Vaysse L. Gregory L. G. Harbottle R. P. Perouzel E. Tolmachov O. Coutelle C. (2006). Nuclear-targeted minicircle to enhance gene transfer with non-viral vectors *in vitro* and *in vivo*. J. Gene Med. 8, 754–763. 10.1002/jgm.883 16532508

[B117] Wang T. Wei J. J. Sabatini D. M. Lander E. S. (2014). Genetic screens in human cells using the CRISPR-Cas9 system. Science 343, 80–84. 10.1126/science.1246981 24336569 PMC3972032

[B118] Weber A. J. Harman C. D. Viswanathan S. (2008). Effects of optic nerve injury, glaucoma, and neuroprotection on the survival, structure, and function of ganglion cells in the mammalian retina. J. Physiol. 586, 4393–400. 10.1113/jphysiol.2008.156729 18565994 PMC2614024

[B119] West S. C. (2003). Molecular views of recombination proteins and their control. Nat. Rev. Mol. Cell Biol. 4, 435–445. 10.1038/nrm1127 12778123

[B120] Wirt S. E. Porteus M. H. (2012). Development of nuclease-mediated site-specific genome modification. Curr. Opin. Immunol. 24, 609–616. 10.1016/j.coi.2012.08.005 22981684

[B121] Woltjen K. (2009). piggyBac transposition reprograms fibroblasts to induced pluripotent stem cells. Nature 458, 766–770. 10.1038/nature07863 19252478 PMC3758996

[B122] Wu Y. Liang D. Wang Y. Bai M. Tang W. Bao S.. (2013). Correction of a genetic disease in mouse via use of CRISPR-Cas9. Cell Stem Cell 13, 659–662. 10.1016/j.stem.2013.10.016 24315440

[B123] Yin H. Kanasty R. L. Eltoukhy A. A. Vegas A. J. Dorkin J. R. Anderson D. G. (2014a). Non-viral vectors for gene-based therapy. Nat. Rev. Genet. 15, 541–555. 10.1038/nrg3763 25022906

[B124] Yin H. Xue W. Chen S. Bogorad R. Benedetti E. Grompe M.. (2014b). Genome editing with Cas9 in adult mice corrects a disease mutation and phenotype. Nat. Biotech. 32, 551–553. 10.1038/nbt.2884 24681508 PMC4157757

[B125] Yusa K. Rad R. Takeda J. Bradley A. (2009). Generation of transgene-free induced pluripotent mouse stem cells by the piggyBac transposon. Nat. Methods 6, 363–369. 10.1038/nmeth.1323 19337237 PMC2677165

[B126] Zhu W. Mao Y. Zhou L. F. (2005). Reduction of neural and vascular damage by transplantation of VEGF-secreting neural stem cells after cerebral ischemia, in Intracranial Pressure and Brain Monitoring XII, Vol. 95, eds Poon W. S. Chan M. T. V. Goh K. Y. C. Lam J. M. K. Ng S. C. P. Marmarou A. Avezaat C. J. J. Pickard J. D. Czosnyka M. Hutchinson P. J. A. Katayama Y. (Vienna: Springer), 393–397. 10.1007/3-211-32318-x_80 16463888

[B127] Zou J. Mali P. Huang X. Dowey S. N. Cheng L. (2011). Site-specific gene correction of a point mutation in human iPS cells derived from an adult patient with sickle cell disease. Blood 118, 4599–4608. 10.1182/blood-2011-02-335554 21881051 PMC3208277

